# Advanced SPARQL querying in small molecule databases

**DOI:** 10.1186/s13321-016-0144-4

**Published:** 2016-06-06

**Authors:** Jakub Galgonek, Tomáš Hurt, Vendula Michlíková, Petr Onderka, Jan Schwarz, Jiří Vondrášek

**Affiliations:** Institute of Organic Chemistry and Biochemistry, Academy of Sciences of the Czech Republic, Flemingovo nám. 2, 166 10 Prague 6, Czech Republic; Faculty of Mathematics and Physics, Charles University in Prague, Malostranské nám. 25, 118 00 Prague 1, Czech Republic

**Keywords:** Resource Description Framework, SPARQL query language, Database of small molecules

## Abstract

**Background:**

In recent years, the Resource Description Framework (RDF) and the SPARQL query language have become more widely used in the area of cheminformatics and bioinformatics databases. These technologies allow better interoperability of various data sources and powerful searching facilities. However, we identified several deficiencies that make usage of such RDF databases restrictive or challenging for common users.

**Results:**

We extended a SPARQL engine to be able to use special procedures inside SPARQL queries. This allows the user to work with data that cannot be simply precomputed and thus cannot be directly stored in the database. We designed an algorithm that checks a query against data ontology to identify possible user errors. This greatly improves query debugging. We also introduced an approach to visualize retrieved data in a user-friendly way, based on templates describing visualizations of resource classes. To integrate all of our approaches, we developed a simple web application.

**Conclusions:**

Our system was implemented successfully, and we demonstrated its usability on the ChEBI database transformed into RDF form. To demonstrate procedure call functions, we employed compound similarity searching based on OrChem. The application is publicly available at https://bioinfo.uochb.cas.cz/projects/chemRDF.

**Graphical Abstract:**

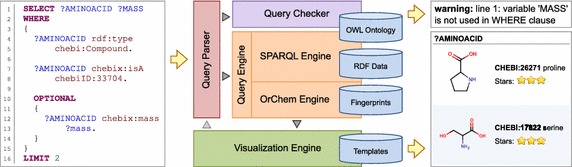

## Background

Databases of small molecules play a key role in many areas of cheminformatics and bioinformatics research and applications. There are many kinds of small molecule databases [1]—one of the most comprehensive is the ChemSpider database, which offers access to approximately 44 million compounds [2]. Some databases are general purpose; others are focused on a specific area of research, such as drugs and their targets or metabolites found in the human body. The vast majority of these databases are publicly available. Many companies build their own proprietary databases as well, which demonstrates the large range of utilization and different foci of such databases [3, 4].

Such databases should fulfill criteria for simple and effective searching. Most small molecule databases assume that users know the name or structure of the compound of interest. The structure or name can then be used as a database query to obtain additional information stored in the database. Alternatively, the search can be based on specifying desired properties of the compound in a fixed query form. These approaches are sufficient in many cases, but they are insufficient if new information needs to be derived from existing data, for example, to find compounds according to their complex interactions with other compounds or biological entities.

For more powerful database searching, a user needs to know the data model used and the corresponding query language. Query languages are typically defined on the logical database level, which describes data organization in terms of the database paradigm used to store data. These can cause confusion for common users, who intuitively think about data on the conceptual database level, which is focused on ontological description of data. For example, one of the most well-known query languages is SQL, which is defined for the relational data model. In this model, data are represented by tables. To be able to write SQL queries, it is essential to know how the data are organized into tables. However, the organization is determined by various requirements and conditions that are mostly irrelevant from a common user’s point of view. To make a search easier and more intuitive, it is appropriate to use a technology for which the differences between the conceptual and logical models are not so distinct. The Resource Description Framework (RDF) and the SPARQL query language can be used in such cases [5]. The RDF model defines organization of data on a logical level, but it is also related to some conceptual modeling approaches.

Data interoperability is another important criterion. Usage of data coming from different data sources can be complicated if the sources describe data in incompatible ways. This issue is also addressed by RDF, which allows description of vocabularies defining unique identifiers and meanings that are used for data description. Moreover, SPARQL supports federated queries; this means that one query can employ data from different sources (SPARQL endpoints) managed by different organizations.

In recent years, the RDF framework and the SPARQL language have become more widely used in the area of cheminformatics databases [6]. Chem2Bio2RDF focuses on linking data from different sources using RDF [7]. Well-known PubChem data are available for download in RDF format, which allows the user to import them into a personal RDF storage and query them using SPARQL [8]. ChemSpider also allows users to download compound data in RDF format [9]. A SPARQL endpoint that allows users to submit queries on ChemSpider data is in development and should be finished soon. In addition, the European Bioinformatics Institute (EMBL-EBI) serves SPARQL endpoints to many of its databases [10]. Another database that uses SPARQL to support advanced searching is the neXtProt database, which focuses on human proteins [11].

In our work, we focus on the use case in which a database is accessible through a web interface that allows the user to submit SPARQL queries. Although the RDF platform is already widely used in many cheminformatics areas, we identify several deficiencies that make usage of RDF databases restrictive or non-user-friendly:

1. Special procedures are not supported. Not all kinds of data can be precomputed simply and stored directly in a database. Instead, they are computed from given parameters on demand. A typical example is a list of compounds that are similar to a given compound. In relational databases, compound similarity is supported by a chemical cartridge [4]. This defines stored procedures called from SQL that return required similar compounds. However, SPARQL does not contain support for calling stored procedures, and many RDF storages lack a proprietary extension. For this reason, similarity searches and other useful functions are not typically supported by chemical databases based on RDF storage.
2. Writing SPARQL queries is prone to errors. A query written in SPARQL may not respect the data ontology but can still be considered valid. This means, for example, that a query can contain an identifier that is not included in the database (and is therefore not described in the ontology) or that it is possible to use identifiers in a nonsense combination from an ontology point of view. In such cases, an empty result is returned, and the user is not informed about the source of the problem. This makes query debugging troublesome.
3. Presentation of results is either stern or inflexible. In the simplest approach, a query result is presented as a table of raw values. In this manner, for example, found compounds are presented only by their database identifiers. Additional information (e.g., compound names or human readable identifiers) have to be retrieved explicitly by the query. This approach is used, for example, by EBI, which uses a general purpose SPARQL endpoint to submit queries. On the other hand, in neXtProt, found entities are presented together with some details. However, the system is limited to present only one class of entities.

In this paper, we present our proposals to develop a small molecule RDF database that addresses these issues. We extended a SPARQL engine to allow querying of data that cannot be stored directly in a RDF database. We designed an algorithm that performs check of a query against data ontology to identify possible user errors. In addition, we introduced an approach to visualize retrieved data in a user-friendly way. To integrate all features of our approaches, we developed a simple web application, and we demonstrated its overall functionality on data retrieved from the ChEBI database [12].

## Resource Description Framework overview

In the RDF data model, information is expressed as simple statements about entities (called resources in RDF terminology) [5, 13]. Each statement is formed as a triple: subject, predicate, and object. The statement expresses a relation between two resources—the subject and the object. Specifically, it expresses that the subject has a property identified by the predicate, which has the object as its value. For public identification of resources, the International Resource Identifier (IRI) is employed. Because IRIs can be very long, namespace prefixes can be defined. The namespace prefix represents an initial section of IRIs that can be replaced by a prefix. Predicates are identified by IRIs as well. A predicate IRI can appear in the subject or object position in other triples, so it is possible to make statements about the property. This is very useful for description of data ontology. If public identification of resource is not needed, blank nodes, which have only local meanings, can be used. Special classes of resources are literals (e.g., text, date, numbers) that express simple property values and can be used only in the object position in a triple.

The Web Ontology Language (OWL) can be used to describe data ontology [14–16]. OWL describes classes of resources and their properties. The most important feature of classes is their subclass hierarchy. Properties are described separately, and their most important features are domains and ranges. Properties also can be arranged in a hierarchy. For example, mass or charge properties can be defined as subproperties of the common chemical property. The ontology itself is stored with regular data in the form of triples. This allows users to query ontology information in the same way as they query regular data.

One of the most important features of RDF databases is the ability to infer triples that are not physically stored in the database. This is especially useful in conjunction with the ontology description. For example, if the resource S has property P1 with value V, and property P1 is subproperty of property P2, then it is inferred that resource S has property P2 with value V. This is particularly important and useful for querying because it allows for simple query construction.

The SPARQL query language has been introduced as a means to query data [17]. The basic concept of the language is based on a triple pattern that has the same form as an RDF triple but can contain variables in arbitrary positions (i.e., in the subject, predicate, or object position). During query evaluation, solution mappings—mappings from the pattern variables to resources for which triples exist in a database—are returned as results. The map between the variable and the value is binding, and the variable is said to be bound to the value. Note that SPARQL allows grouping of multiple triple patterns that have common parts into a single pattern. Patterns can be combined to express more powerful and complex patterns. For example, the union pattern can be used to describe variants; the optional pattern describes parts that are not mandatory; and the minus pattern subtracts some unwanted solutions. SPARQL 1.1 also introduces property paths, which extend classical triple patterns. This allows combination of multiple predicates into one triple pattern. Single predicates represent binary relations between resources. These relations can be combined into complex relations by various operators, including the inverse path operator (denoted by the symbol ^ and defined as the inverse relation), the sequence path operator (denoted by / and defined as the composition of binary relations), the alternative path operator (denoted by | and defined as the union of binary relations), and the one or more path operator (denoted by + and defined as the transitive closure of a relation).

In most cases, RDF data are managed and processed by complex frameworks. The most widely used frameworks are Apache Jena [18] and Sesame [19]. These frameworks contain their own RDF storages. Other storages typically contain providers that allow connections to be made through these frameworks. RDF storages are often based on relational databases (Oracle [20], OpenLink Virtuoso [21]) or focused directly on storing RDF triples (Ontotext GraphDB [22]).

Most RDF storages do not support calling general procedures from SPARQL queries. The most general mechanism to call procedures is included in Jena. This extension is called property functions, and it allows to call special procedures to retrieve data by methods other than the usual pattern matching [23, 24]. In a SPARQL query, a property function is represented by a special IRI that is registered in the Jena SPARQL engine. If the IRI is used in the predicate position of a triple pattern, the registered Java method is invoked by the engine, and the subject and the object of the triple pattern are passed as the method parameters. Based on the parameters, the methods return solution mappings that represent a result of the triple pattern.

## Implementation

To develop our proposed system, we decided to use OpenLink Virtuoso [21], a database server with very good performance that is used by various bioinformatics and cheminformatics projects. However, Virtuoso does not support calling general procedures from SPARQL queries. Although this support is included in Jena, its performance is not sufficient for our purpose.

### Procedure call extension

We designed our own extension to call procedures from SPARQL queries. One of the main requirements for the procedure call extension was that it should not change the syntax of the SPARQL language. This requirement is important for the possibility to use third-party components that work with SPARQL queries without modifying these components. Thus, only existing valid triple pattern syntax can be used to call a procedure. We also require only minimal changes in semantics. The extension should have no or minimal effect on existing language constructs. Also, the semantics should be maximally transparent for users; results returned by a procedure call should be the same as in the case in which data are stored in the database and triple patterns representing the procedure call are regular patterns.

To avoid affecting the syntax, we used an approach very similar to that used in Jena [23]. A procedure call is expressed as a triple pattern using a special predicate IRI that identifies the procedure. The advantage of this approach is that it is transparent from a user’s point of view. It works the same way as in the case that triples representing procedure parameters and corresponding results are stored in the database. In our solution, the object of the triple pattern always represents parameters, and the subject of the triple pattern always represents results of the procedure call. However, the way the parameters are passed is a little bit different than in Jena. In our approach, the object of the triple pattern representing the procedure call must be a blank node expressed in abbreviated form (i.e., by using []). Properties of the blank node are then understood as procedure parameters. Objects of these properties have to be constant values or bound variables. During evaluation, these values are then passed into the procedure as parameters. IRIs of the parameters are part of the procedure call definition. If a specified property is not used inside blank node abbreviated form, the default value can be used (if it is specified).

To pass multiple parameters from the object positions, Jena allows use of RDF collections (i.e., a list of resources enclosed in parentheses). Compared with Jena, our approach has several advantages. Passed parameters are always connected with their names (with property IRIs of the blank node). This allows specification of parameters in arbitrary order, definition of default values of parameters, and better descriptions of parameters by database ontology.

**Fig. 1:**
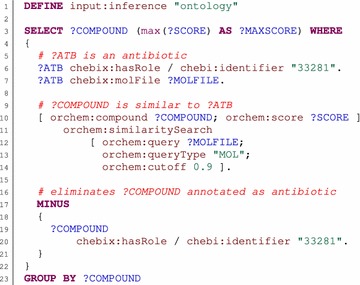
SPARQL query using similarity search. This example SPARQL query uses a procedure call named *orchem:similaritySearch* to identify compounds that are similar to a given structure. The task of the query is to select all compounds that are not annotated as antibiotics, but that are similar to a compound that is annotated as an antibiotic. In addition to the compounds, the query also returns similarity scores to the most similar antibiotics. The first triple pattern (*line 6*) binds the *ATB* variable to compounds that are annotated as antibiotics (identified by ChEBI ID *33281*). The following triple pattern (*line 7*) binds the *MOLFILE* variable to the MOL structures of these compounds. The procedure call is identified by the *orchem:similaritySearch* IRI and is represented by the triple pattern on *lines 10–14.* The blank node used in the object position (*lines 12–14*) represents parameters of the procedure call. The query structure is denoted by the *orchem:query* IRI, and its value is specified by *MOLFILE*. Other parameters are constant. The type of the query structure is denoted by the *orchem:queryType* IRI, and the cutoff similarity score is denoted by the *orchem:cutoff* IRI. The blank node used in the subject position (*line 10*) represents multi-value results of the procedure call. The *COMPOUND* variable is bound to the similar compounds found (identified by the *orchem:compound* IRI), and the *SCORE* variable is bound to their appropriate similarity score (identified by the *orchem:score* IRI). The minus pattern (*lines 17–21*) eliminates all identified compounds (to which *COMPOUND* is bound) that are annotated as antibiotics. Finally, the results are grouped by *COMPOUND* (*line 23*), and the compounds (*COMPOUND* variable) and their maximal similarity scores to some antibiotics (*MAXSCORE* variable) are returned as the final result (*line 3*)

**Fig. 2:**
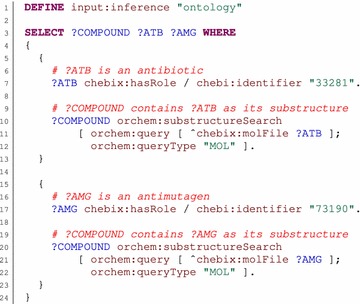
SPARQL query using substructure search. This example query demonstrates how to select compounds that contain an antibiotic and an antimutagen as substructures. The first triple pattern (*line 7*) binds the *ATB* variable to compounds that are annotated as antibiotics (identified by ChEBI ID *33281*). Substructure search is then used to determine compounds that contain the given *ATB* compounds as substructures. The procedure call is identified by the *orchem:substructureSearch* IRI and is represented by the triple pattern on *lines 10*–*12*. The blank node used in the object position (*lines 11*–*12*) represents parameters of the procedure call. The query structure is denoted by the *orchem:query* IRI, and its value is specified by another blank node, which represents the structure of a compound to which the *ATB* variable is bound. The type of the query structure is denoted by the *orchem:queryType* IRI. The *COMPOUND* variable is bound to the identified compounds. Compounds that contain an antimutagen (identified by ChEBI ID *73190*) are identified in the same way (*lines 17*–*22*). Because the results of both procedure calls are represented as the *COMPOUND* variable, the variable contains the intersection of the procedure call results at the end. The query returns the identified compounds (*COMPOUND* variable) together with the appropriate antibiotic (*ATB* variable) and antimutagen (*AMG* variable) as the final result (*line 3*)

Results of a procedure call are represented by the subject of the triple pattern. In a simple case, the subject is a variable that is bound to procedure results. In some cases, it can be useful to be able to return structured result values consisting of more individual values (e.g., compounds that are similar to the given query structure along with their similarity scores). To make this possible, we defined a multi-value form of a procedure call. We used an approach similar to that used to pass parameters of procedures. In this form, the subject of the procedure call pattern is also expressed as a blank node in abbreviated form. Individual properties of the blank node then identify result value components. Objects of such properties are typically variables that are bound to result values during evaluation of the procedure call. If the object of a property is constant, it works as a filter on result values. Examples of complex queries using procedure calls are shown in Figs. 1 and 2.

Although the syntax of the language is not affected, to add procedure call support, the parser of the language has to be extended to recognize procedure call patterns (together with associated blank nodes expressed in abbreviated form) and process them in a special way. To extend the language semantics, one only needs to extend the way in which the so-called GraphGraphPattern (i.e., graph that sequentially contains other patterns) is processed [17].

Moreover, the proposed extension has to be sufficiently flexible and general-purpose. For this reason, a set of supported procedure calls cannot be fixed and hardwired into the source code of the extension. Instead it is configured by a configuration file that describes the procedure calls. It allows adding support for new procedure calls into a system without changing the extension source code.

### Algorithm for procedure call translation

Although the semantics are minimally affected, we decided not to modify the existing Virtuoso code. Instead, we used the fact that Virtuoso can combine SQL and SPARQL queries and designed a preprocessor that translates extended SPARQL queries into SQL/SPARQL. If a query does not contain a procedure call, the query is translated directly into SPARQL language. Otherwise, parts are translated into SPARQL where possible, and rest of the query is translated into SQL. We tried to keep as many parts as possible in SPARQL, which allows Virtuoso to make SPARQL optimizations. The translation phase is straightforward. For a given query, the parser produces a syntax tree that represents the query according to SPARQL grammar. It also identifies procedure call patterns and removes syntactic sugar. Our parser is based on ANother Tool for Language Recognition (ANTLR) [25]. The syntax tree is translated from the leaves to the root. If all subnodes of a tree node are translated into SPARQL, the node is translated into SPARQL; otherwise, it is translated into SQL. An exception is a node representing a procedure call, which is always translated into the appropriate SQL procedure call specified in the configuration file.

### Ontology checking of queries

Based on the ontology, the most important following potential errors can be checked:

1. Existence of a property IRI. If the IRI used in the predicate position of a triple pattern does not exist in the ontology, the warning is emitted, because such a triple pattern has no solution if we assume that data are fully described by the ontology.
2. Correctness of a literal value. The predicate IRI of the triple pattern can be used to determine the range of the property. This information then can be used to check whether constant literal value in the object position is an instance of the appropriate range class.
3. Correctness of a property path. Individual parts of a property path have to be correctly interconnected. This means that the range of one part and the domain of a following part cannot be disjoint classes. Otherwise, the triple should have no solution.
4. Consistency of used variables. If a variable is used in a triple pattern, the ontology can be used to determine to which class of resources the variable will be bound. Information determined for the variable from other parts of the query has to be consistent; otherwise, the query has no solution.

Checking for the first three error types is simple because they are local; no query context and no other information except the ontology are needed to perform such checks. Checking the consistency of used variables (error type no. 4) is more complicated because information obtained from various parts of a query has to be taken into account. If a triple pattern contains a variable in the subject or object position, the ontology can be used to obtain information about the class of resources to which variables will be bound. Information retrieved from different parts of the query and relating to the same variable has to be consistent. As an example, assume that a query employs a group pattern that contains two triple patterns using the same variable. If the first triple pattern contains the variable in the subject position, we can use the predicate IRI of the triple pattern to obtain the domain of the property identified by the IRI. This domain specifies the class of resources to which the variable should be bound. If the second triple pattern contains the variable in the object position, we can use the predicate IRI of the triple pattern to obtain the range of the property, also specifying the class of resources to which the variable can be bound. If these two classes are denoted as disjoint by the ontology, then it logically follows that the query cannot have a solution, because the query does not make sense with respect to the given ontology. Nevertheless, relative positions of variable occurrences have to be taken into account. This can be demonstrated by a case in which the same variable occurs in different branches of a union pattern; these occurrences are independent, and thus they cannot ever be considered a source of conflict.

### Variable consistency checking algorithm

The variable consistency checking algorithm works with classes of resources to which variables can be bound. Basic classes are represented by IRIs and described by the ontology stored in a database. A query can contain complex patterns, therefore the basic classes may not be sufficient to describe classes of resources to which variables can be bound. For this reason, the algorithm has to be able to work with unions and intersections of the classes. Two classes are considered to be consistent if they are not disjoint classes. For basic classes, the information about whether the classes are disjoint is obtained directly from the ontology according to the values of the *owl:disjointWith* property. Otherwise, the consistency of the classes is checked recursively according to the union and intersection operators.

Briefly, the algorithm checks whether information obtained for a variable from one specific position in the checked query is consistent with information obtained for the same variable from different parts of the query. The algorithm works with the syntax tree of the checked query that is produced by the same parser that is used for query translation. For each node of the syntax tree, two kinds of information are recursively computed:

- The class registry contains maps between variable names and classes of resources to which variables can be bound (which are denoted classes of the variables). The registry represents classes of resources that are inferred from the whole pattern represented by the node.
- The location registry collects all variables and their locations that occur in the pattern that have to be checked for consistency. Together with the name of the variable and its location in the query, the class of the variable inferred from the variable occurrence is also stored.

In general, for a given variable name, the algorithm checks the consistency between variable classes stored in the location registry and classes stored in the class registry that are derived from the other parts of the checked query. Nodes of a syntax tree are processed recursively from leaves to the root:

- A triple pattern is always located in a leaf of the syntax tree and presents a source of all class information. If the predicate of the triple pattern is not variable (i.e., it is a property IRI or a property path containing IRIs), then the property IRI is used to obtain the range and domain of the property. If the triple pattern contains a variable in the subject position, the class of the variable is set according to the obtained domain. And vice versa, if the triple pattern contains a variable in the object position, the class of the variable is set according to the obtained range. A triple pattern using the *rdf:type* predicate and having a variable in the subject position is a special case denoting that the object is the class of the variable. If the pattern contains a variable in the predicate position, the class of the variable is *rdfs:Property*. All obtained class information is stored in the class registry of the node. Similarly, positions of variables used in the triple and their classes are stored in the location registry of the node.
- A group pattern collects patterns (enclosed in braces) that have to all be fulfilled. It is the main place where the consistency of class information is checked. For each child pattern of the processed group pattern, the class registries of all other child patterns are merged by the class intersection operation. For each variable stored in the location registry of the child pattern, class consistency between the class of the variable and the class of the variable stored in the merged class registry is checked. If the classes are inconsistent, a warning about incorrect usage of the variable in the given location is reported. After the check phase, the class registries of all child patterns are merged by the class intersection operation into the class registry of the processed group pattern, and the child location registries are merged into the location registry of the processed group pattern.
- A union pattern joins two child group patterns that represent independent alternatives. Class registries of the child group patterns are merged by the union class operation into the class registry of the processed union pattern. Location registries of child patterns are normally merged into the location registry of the processed pattern.
- An optional pattern includes only one group pattern as the child pattern that is considered an optional part of a query. Because the optional pattern is not mandatory, its class registry is kept empty. Its location registry is set according to the location registry of its child pattern, because variables used in the optional pattern still have to be consistent with the rest of the query.
- A minus pattern also includes only one group pattern as the child pattern. It eliminates query solutions that are not compatible with solutions of the child pattern. For this reason, registries of minus patterns are kept empty.

**Fig. 3:**
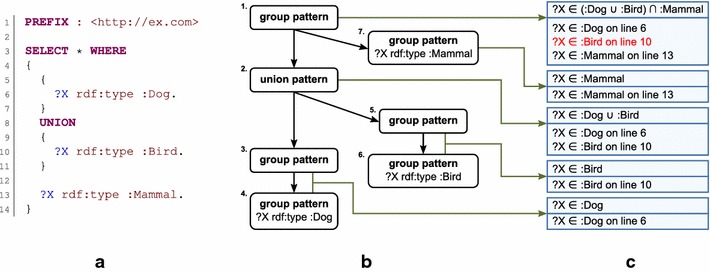
Example of variable consistency check. This example demonstrates the work of the variable consistency checking algorithm. Part **a** shows a simple SPARQL query intended to select resources that belong to the *:Dog* class or to the *:Bird* class and also belong to the *:Mammal* class. The query has a solution in standard animal ontology. However, the second alternative of the union pattern is inconsistent with the rest of the query (because birds are not mammals). Therefore, this alternative has no influence on the final result, and the user should be warned. Before checking, the query is transformed into a syntax tree by the parser. The relevant part of the parser tree is shown in Part **b**. For better clarity, *nodes* are identified by numbers. The tree is then processed by the checking algorithm from the leaves to the root. For *each node*, the class registry and the location registry are computed. Contents of the registries are shown in *boxes* in Part **c**. The *top part* of *each box* contains the class registry, and the *bottom part* of the *box* contains the location registry. A *green arrow* indicates for which node registries were computed. *Triple patterns 4*, *6*, and *7* simply denote requested classes of variable *X*, and this information is written into their registries. *Group* *patterns* *3* and *5* contain only one child pattern, so their registries are the same as registries of these child patterns. After that, *union* *pattern* *2* merges information from *group* *patterns 3* and *5*. Finally, *group* *pattern* *1* is processed. This pattern contains two child patterns, so the checking phase has to be performed. The location registry of *node* *7* is compared with the class registry of *node* *2*. Class *:Mammal* is consistent (is not disjoint) with the union of *:Dog* and *:Bird* classes; thus, this part is correct. And vice versa, the location registry of *node* *2* is compared with the class registry of *node* *7*. Class *:Dog* is consistent with *:Mammal*, but class *:Bird* is not consistent with *:Mammal*. Therefore, the warning is generated for variable *X* used on *line 10*

An example of how the variable consistency of a query is checked is shown in Fig. 3.

### Presentation of results

The most important requirement for the presentation layer is that it should not be hardwired directly into the code. The basic idea is that descriptions how instances of classes should be visualized are stored in the database together with the ontology describing the classes. For practical usage, the visualization of a class is described in a selected template language, and only the name of the template is stored in the database. In our project, we used a simple template language called Apache Velocity [26]. The task of the template is to generate HTML code that represents the given instance of the class for which the template is intended. Velocity templates can contain static text (HTML code in our case) that is directly passed into the output during template evaluation by the Velocity template engine. The important parts of a template are references that allow representation of dynamic content, i.e., content that is specific for the represented instance. Velocity has three types of references. The basic type of references are Velocity variables (prefixed by a dollar sign) that can refer to Java objects. References can have reference properties that represent other type of references. A property is connected by the dot symbol with a reference and represents the value of an appropriate Java getter method called on the reference. Another type of references is called methods, which are similar to reference properties but contain additional arguments enclosed in parentheses. The resource that should be presented by a template is stored in a reference that is set outside the template engine before the template evaluation is started. The final important parts of the Velocity template language are directives that control the process of output generation. Directives include mechanisms to express conditions, loops, variable assignments, and others. All directive names are prefixed by a number sign (#). There are two types of directives. A line directive is parametrized by data enclosed in parentheses. A typical example is the *#set* directive, which assigns a value to a Velocity variable. In addition to parameters, a block directive contains also a body enclosed between the directive name and *#end*. For example, the body of the *#foreach* directive is repeated according to the parameters of the directive. The possibility to define new directives is an important feature of Velocity. To allow presentation of instances by Velocity templates, we define two special new directives:

- The *#sparql* directive is a block directive that allows submission of a SPARQL query to obtain information from the database. The query is written in the body of the directive. The result of the query is stored in a Velocity variable that is used as a parameter of the directive and that represents all results of the query. The Velocity variable can be looped over for all individual results using the *#foreach* directive. Each individual result has reference properties that correspond to SPARQL variables used in the query.
- The *#url* directive is a block directive that allows generation of a hyperlink to a resource. The output of a template is used inside our web application, and thus, it should not contain a regular HTML link. Instead, the *#url* directive generates a special code that informs the application that a view of the new resource is requested. The directive is parametrized by the linked resource, with the body containing the text of the link.

**Fig. 4:**
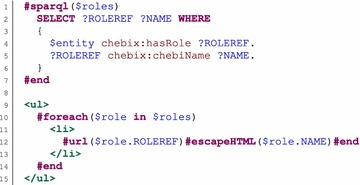
Example of a velocity template. For a given compound, the example template generates an HTML list of its chemical or other roles. Each item in the list is a hyperlink to the represented role. The visualized compound is referenced by the *$entity* Velocity variable that is set by the system before the template is evaluated. The body of the *#sparql* directive contains a query that returns the roles of the compound and their names. The query is parametrized using the *$entity* reference. Before the query is submitted, the reference is replaced by its value. The query results are returned by the database engine and stored in the *$roles* Velocity variable specified as a parameter of the directive. Individual results are processed by the *#foreach* directive. It sequentially stores a result into the *$role* Velocity variable and processes its body. The *$role* Velocity variable is used to access values of SPARQL variables; the reference to the role entity (*$role.ROLEDEF*) and to the name of the role (*$role.NAME*) are accessible inside the body. These references are used by the *#url* directive to produce links to the roles. The *#escapeHTML* macro escapes characters in the name value to be placed into HTML

An example of usage of these directives is shown in Fig. 4.

### Web application

In the previous sections, we described suggested approaches for database querying. To examine their applicability, we developed a simple web application that allows users to submit queries and visualize results. The application was developed using Google Web Toolkit (GWT) [27]. The user interface of the application is divided into three parts. The left part contains a query editor that allows users to write SPARQL queries. We used the third-party component CodeMirror [28] as the editor, which is interconnected with our checking algorithm. During query typing, the editor sends the written query to the server for checking, and errors and warnings are immediately reported in the editor.

The query result is visualized as a table in the central part of the application. Each variable used in the select clause of a query is represented by one column. Individual solution mappings forming a single result are represented by rows. If the value to which a variable is bound is a resource represented by an IRI or a blank node, the appropriate item template is used to visualize the cell. Otherwise, the value itself is used as cell content.

The right part of the application is used to visualize details about the selected resource. The IRI of the resource can be entered directly by the address bar, or the resource can be selected from the result table. If details about the resource are requested, the application uses the appropriate page template to generate details about the resource.

The application uses two kinds of templates that are specified by separated properties. Both properties use the prefix *template:* (see Table 1). The item template is used to generate small items in the result table, and it is denoted for a class by using the *template:itemTemplate* property. The page template is used for the detailed presentation and is denoted by the *template:pageTemplate* property.

**Table 1:** Namespace prefix definitions

Prefix	Value
chebi	http://bioinfo.uochb.cas.cz/0.9/chebi#
chebix	http://bioinfo.uochb.cas.cz/0.9/chebix#
chebiID	http://bioinfo.uochb.cas.cz/0.9/chebi/
template	http://bioinfo.uochb.cas.cz/0.9/template#
orchem	http://bioinfo.uochb.cas.cz/0.9/orchem#
rdf	http://www.w3.org/1999/02/22-rdf-syntax-ns#
rdfs	http://www.w3.org/2000/01/rdf-schema#
owl	http://www.w3.org/2002/07/owl#
xsd	http://www.w3.org/2001/XMLSchema#

The table shows definitions of all namespace prefixes used in our project. The prefixes are already defined in our SPARQL query engine, and therefore they need not be explicitly defined in SPARQL queries

## Results and discussion

For application of our proposed system, it is necessary to select and load data into the database, define their ontology, write templates for data visualization, and define stored procedures that can be called from SPARQL queries.

### Transformation of the ChEBI database

We decided to use the ChEBI database as a data source. ChEBI is sufficiently large for our purposes, and we have previously performed an analysis of this database [29]. The ChEBI data are available in a relational database form, and thus the data needed to be converted into the form of triples. For this purpose, we defined ad hoc ontology to demonstrate our proposed approaches. We used the prefix *chebi:* as the base of IRIs used by the ontology (see Table 1). The main entities in the ChEBI database are compounds that are stored in the *COMPOUNDS* table. Each compound stored in ChEBI is represented as an instance of the *chebi:Compound* class in our database. IRIs that identify compounds use the prefix *chebiID:* (see Table 1).

ChEBI stores features of compounds in separate tables. These features include names (*NAMES* table), basic chemical features (*CHEMICAL_DATA* table), accessions to other databases (*DATABASE_ACCESSION* table), and chemical structures (*STRUCTURES* table). The tables describing features contain not only the feature values themselves but also other related information. All tables contain names of sources from which the original values were taken. For this reason, ChEBI features are not represented directly as literal values but as instances of appropriate classes that are subclasses of the *chebi:CompoundProperty* class. These instances are connected with respective compounds by appropriate properties that are subproperties of the *chebi:hasProperty* property. Each such resource representing a ChEBI feature has an appropriate property suffixed by *Value* representing a value of the ChEBI feature. Moreover, the resources also have the common property *chebi:source*, which determines the name of the original data source. Other properties can be added depending on the type of feature.

Chemical relations between compounds are stored in the *RELATION* and *VERTICE* tables. In our database, they are represented in a similar way as other features. The chemical relations are represented as instances of appropriate classes that are subclasses of the *chebi:CompoundRelation* class. These resources are connected to the respective compounds by appropriate properties that are subproperties of the *chebi:inRelationWith* property. A related compound is specified by the *chebi:inRelationWithValue* property. In this case, other properties can also be specified for resources representing chemical relations.

**Fig. 5:**
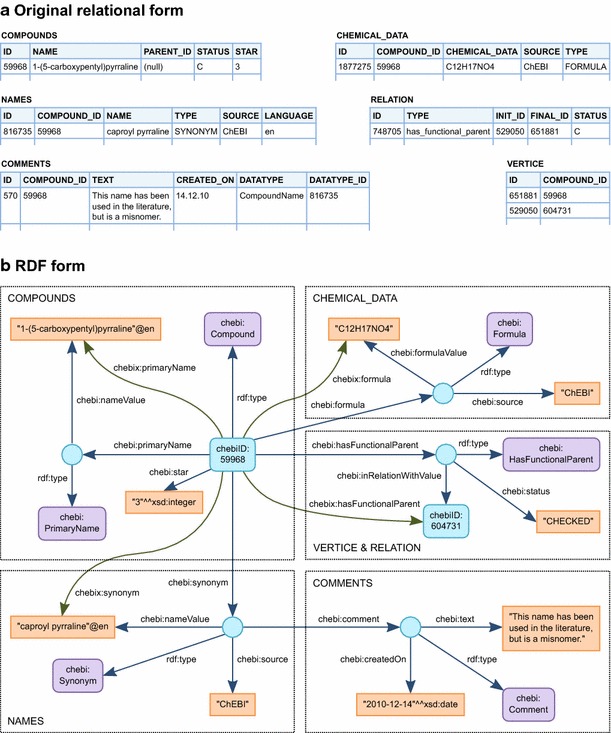
Conversion of ChEBI into RDF form. This example demonstrates the conversion of the ChEBI database from relational form into RDF form. Part **a** shows selected information about the ChEBI entity with ID *59968*. Part **b** shows the same information coded in RDF form. For better clarity, it is represented as a connected graph. *Each triple* is represented as an arc labeled by the predicate IRI leading from the subject to the object. *Blue arcs* represent triples stored directly in the database. *Green arcs* represent triples inferred by property chains. Literals are represented as *boxes*, IRIs as *rounded* *boxes*, and blank nodes as *circles*. *Dotted line boxes* are not parts of the RDF model; they only highlight which table of the relational form was used to generate triples. In relational form, each table contains a primary key named *ID*. The basic data about the compound are stored in the *COMPOUNDS* table. In RDF form, the compound is identified by IRI *chebiID:59968*, and belongs to the *chebi:Compound* class. The basic data are converted directly as appropriate properties (e.g., *chebi:star*) of the compound. An exception is the compound name, which is converted as an instance of *chebi:PrimaryName*. The reason for this is compatibility with representations of other compound names. Other names of compounds are stored in the *NAMES* table. The *COMPOUND_ID* is a foreign key that refers into the *COMPOUNDS* table. The *TYPE* column lists the type of the name. In this case, the name is coded as an instance of the *chebi:Synonym* class. Comments are stored in the *COMMENTS* table. The *DATATYPE* column indicates which data are commented. In this case, the *DATATYPE* value is *CompoundName*, and thus the *DATATYPE_ID* is a foreign key into the *NAMES* table. As an example of other features, the *CHEMICAL_DATA* table is employed. Similarly as for the *NAMES* table, *COMPOUND_ID* is a foreign key into the *COMPOUNDS* table, and the *TYPE* column indicates the type of the stored data. Therefore, the example chemical data is converted as an instance of the *chebi:Formula* class. Relations between compounds are stored in *RELATIONS* tables. Types of relations are listed in the *TYPE* column. Related compounds are specified indirectly. The *RELATIONS* table contains foreign keys (*INIT_ID* and *FINAL_ID*) that refer to the *VERTICE* table that translates vertex IDs into the appropriate related compound IDs. In this example case, the relation is coded as an instance of *chebi:HasFunctionalParent*

The ChEBI database also contains comments (*COMMENTS* table) that are assigned to compounds or features. These comments are represented as instances of the *chebi:Comment* class and are connected with the commented resource by the *chebi:comment* property. An example showing the basic concept of the conversion is shown in Fig. 5.

An approach in which ChEBI features and relations are represented as instances of classes has the advantage that additional information (source name, comments, etc.) can be connected. On the other hand, in a SPARQL query, two properties are needed to obtain a feature value. If a user wants to obtain the value of a ChEBI feature of a compound, it is necessary to use a property connecting the compound with the resource representing the ChEBI feature, and then to use the property connecting this resource with the feature value. To solve this minor issue, we defined a property chain that allows the connection of these two properties to be represented as a single property [16]. The name of the chain property is the same as the name of the first property in the chain but with the prefix *chebix:* (see Table 1) instead of *chebi:*. Usage of property chains is also shown in Fig. 5.

The property chains are defined in the ontology. Unfortunately, Virtuoso does not contain support for property chains. We therefore extended our translation algorithm to expand property chain IRIs that occur in the predicate position into the property path using the specified property chain properties. This cannot be considered full property chain support, but it is suitable for basic usage.

Images of chemical structures were generated from Structure-Data Files (SDFs) stored in the ChEBI database with the molconvert tool [30].

### Templates

**Fig. 6:**
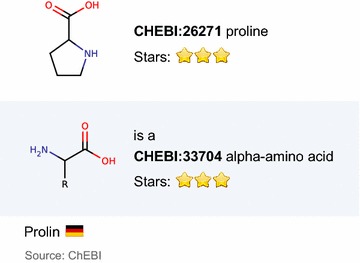
Visualizations generated by item templates. The example shows three visualizations generated by item templates. At the *top* there is the visualization of the compound identified by IRI node *chebiID:26271*. Visualizations of blank nodes that represent the values of compound properties *chebi:isA* and *chebi:synonym* are at the *middle* and at the *bottom*, respectively

Our application is constructed so that for each class (or its superclass), two kinds of Velocity templates are defined—the item template for the result table and the page template for the details. For compounds, the item template shows the compound name together with a small image of the compound structure and a 3-star status that indicates whether the compound has been checked manually by the ChEBI team. For ChEBI compound features, the value and source of the feature value are generated by the item template. An example of visualizations generated by the item templates is shown in Fig. 6. Page templates generate more details, similar to the ChEBI web server. For ChEBI compound features, the details for the associated compound are generated, and the value of the feature is highlighted.

In addition to ChEBI data, our database also contains ontology data. For this reason, it is also necessary to define visualization templates for these data. Item templates visualize classes and properties by their names specified by the *rdfs:label* property and by their IRIs. Page templates generate various detailed information about their hierarchy and usage.

### Use of a chemical cartridge

A large number of chemical cartridges are suitable for our purpose. We decided to use OrChem, which is also maintained by EBI [31]. OrChem supports similarity searches and substructure searches. Both are supported as specific SQL stored procedures. We mapped these procedures directly on our system. All IRIs employed for this mapping used the *orchem:* prefix (see Table 1).

The similarity search procedure (*orchem_simsearch.search*) is represented by the *orchem:similaritySearch* property. Its only mandatory parameter is *orchem:query*, which specifies the structure used as the query structure. The *orchem:queryType* parameter specifies a type of query structure; the default value is *SMILES*, which denotes Simplified Molecular Input Line Entry Specification [32]. Another supported value is *MOL*, denoting an MDL mol file [33]. The remaining parameters restrict the size of the result set. The cutoff similarity score is specified by the *orchem:cutoff* parameter (the default value is 0.8). The maximum number of results can be set by the *orchem:topn* parameter (the default value is −1, which means unlimited). Results of the procedure have multiple values. Identified compounds are denoted by the *orchem:compound* property, and the appropriate similarity score is denoted by the *orchem:score* property. An example is shown in Fig. 1. There is also a simplified variant of the procedure, called *orchem:similarCompoundSearch*. This uses the same set of parameters but returns identified compounds directly and not as multi-value results.

The substructure search procedure (*orchem_subsearch.search*) was mapped in a similar way and is represented by the *orchem:substructureSearch* property. It uses the parameters *orchem:query*, *orchem:queryType*, and *orchem:topn*, which have the same meanings as in the previous case. Moreover, the *orchem:tautomers* parameter indicates whether the query structure should be expanded to its tautomers, and the *orchem:exact* parameters control whether only structures that are equal to the query structure should be returned. The default values of these additional parameters are false. Identified compounds are returned directly as the subject of the procedure call pattern. An example is shown in Fig. 2.

## Limits of the implementations

Although our proposed simple web application works satisfactorily well, we identified several weaknesses.

### Procedure call extension

One of the main requirements for implementation is that usage of procedure calls should be transparent from the user’s point of view. Employing triple patterns with special meanings fulfills this requirement in general. However, there are still situations in which this is not entirely true.

**Fig. 7:**
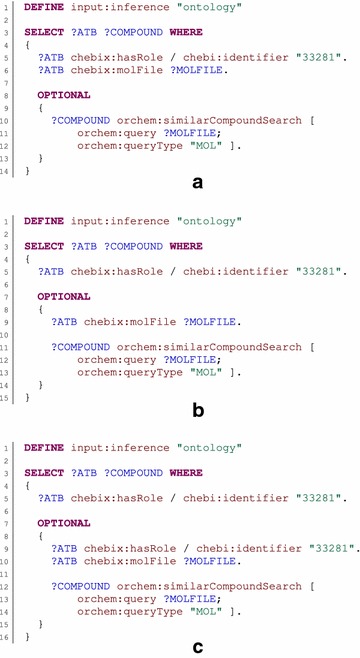
Limits of procedure call semantics. The example queries demonstrate a limitation of procedure call parameters passing. In general, all example queries should select compounds that have the role identified by identifier *33281*, and optionally should find similar compounds for each compound. Although procedure calls should be transparent from the user’s point of view, there is the requirement that variables used as parameters of the procedure call must be bound to values in the same group pattern before the procedure call is evaluated. For this reason, Query **a** is not correct, and the translator will report an error, because the *MOLFILE* variable is used as a parameter but is not bound in the same group pattern. It would seem that Query **b** solves this issue. However, in this case, variable *ATB* is not restricted in the group pattern containing the procedure call, so the procedure will be called for all structures in the database, which is very inefficient. Thus, although some patterns are repeated, only Query **c** can be considered a proper query

The major weakness of our procedure calls support is that variables used as parameters have to be bound to values before a procedure call is executed. This requirement is a consequence of the manner in which patterns are evaluated. In general, a pattern is evaluated independently of the context in which it occurs, and the result of the pattern evaluation is combined with results from other patterns. For this reason, the parameter values can only be taken from patterns that occur before the procedure call pattern in the same group of patterns. This requirement can be a bit counterintuitive in some cases, as illustrated in Fig. 7.

A minor weakness is that the syntax of procedure calls requires usage of the abbreviated blank node form to denote parameters or multi-value results. In this context, if the abbreviated form is expanded into multiple triples by using the label blank node form (or by using a variable), the parser is unable to identify these triples as one procedure call and reports an error message. Another minor weakness is that a triple pattern is interpreted as a procedure call only if the predicate of the triple pattern is the specific procedure call IRI. This means that it is not possible to use this IRI as part of a complex property path. Binding a variable to this IRI and using this variable as the predicate also does not work.

### Ontology checking of queries

**Fig. 8:**
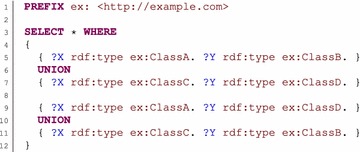
Limits of the variable consistency checking. This example query shows a marginal case in which the query is considered correct by the checking algorithm, but nevertheless, it can be deduced that it has no solution. Consider that all classes used in the example are mutually disjoint. For both union patterns, our checking algorithm observed that the class of variable *X* is the union of the *ex*:*ClassA* and *ex:ClassC* classes. Variable *Y* is handled in a similar way, and thus, the query is considered correct. However, it can be logically deduced that the query has no solution. The first union pattern denotes that the class of variable *X* is *ex:ClassA* only if the class of variable *Y* is *ex*:*ClassB*. Concurrently, the second union pattern denotes that the class of variable *Y* is *ex:ClassB* only if the class of variable *X* is *ex:ClassC*. However, classes *ex:ClassA* and *ex:ClassC* are disjoint, so the query cannot have a solution

Checking focused on the consistency of the variables used can identify many potential problems. However, there are still some marginal cases for which no warning is reported, but it is possible to prove that the query cannot have a result for a given ontology. Our approach checks the usage of a variable independent of other variables. Therefore, it cannot express the fact that instances to which the variable is bound belong to one class only, while instances to which another variable is bound belongs to another class. Such a case is illustrated in Fig. 8. The query is considered to be correct; nevertheless, it is possible to logically infer that the query has no solution for the given ontology.

### Presentation of results

Although the Velocity templates work well for our purpose, we noted several limitations. Because Velocity contains no special support to produce dynamic HTML content, the output of a template is typically static HTML code. This means that all steps needed to produce the visualization of the given resource are produced by the Velocity engine on the server side during template evaluation. As a consequence, there is no simple way to write a template that shows only basic information and allows for extended information to be loaded from the server upon user request. This poses a problem, especially in cases in which details about a resource with a huge number of properties should be generated. However, in general, presentations of other chemical databases are also static. In the future, this issue can be solved by using a template engine that will support creation of interactive content. Nevertheless, for the pilot implementation of our approaches, we decided to select a simple engine that is sufficient for our purposes.

Potentially, a number of queries necessary to produce visual representations may also be a bottleneck. When the result of a user query is returned by the database, the application cannot assume that all resources to which a variable is bound belong to the same class. Thus, for each of these resources, the query to obtain its class and appropriate template name has to be performed. Because the obtained templates are evaluated independently, the queries included in the templates are also performed independently and cannot be grouped. However, this is not an issue for the current data used, and we did not observe any performance problems caused by the queries submitted from the templates.

## Conclusions

We have developed a database system based on RDF technologies. In recent years, these technologies have become more widely used in bioinformatics and cheminformatics research. However, we discovered some issues with these databases from the user’s point of view. Our proposed system addresses the following issues:

1. Most RDF-based systems lack support for special procedure calls. In cheminformatics research, compound similarity search procedures are typical examples. In our system, SPARQL queries can contain procedures to solve such special tasks.
2. SPARQL queries are prone to user errors, because queries do not respect the data ontology. In such cases of user error, the queries return no data but are considered correct, and no warnings are reported. For this reason, we developed an algorithm checking whether a query can have a solution in the given data ontology. The user is warned about potential errors, which makes query writing more comfortable.
3. Many systems use a general presentation layer that presents obtained results without much detail. Eventually, they present some details, but only for the selected classes of resources. In our approach, we developed a general template-based approach allowing presentation of results in a user-friendly manner.

All of these approaches are integrated into a simple web application, available at https://bioinfo.uochb.cas.cz/projects/chemRDF. The application uses data derived from the ChEBI database and employs special similarity search procedures based on OrChem.

Although the need to learn SPARQL language and related technologies can take some time, in return, the user obtains the possibility to submit very powerful queries. The main purpose of our application is not to submit a simple query similar to full-text searching, as in some other databases. We assume that usage of our application will be focused on writing complex queries to solve interesting tasks.

## Availability and requirements

- **Project name:** SPARQL for Chemoinformatics.
- **Project home page:**https://bioinfo.uochb.cas.cz/projects/chemRDF.
- **Operating system(s):** Platform independent.
- **Programming language:** Java.
- **Other requirements:** Modern web browser (tested with Firefox ESR 38, Chromium 46, and Internet Explorer 11).
- **License:** GNU AGPL v3.
- **Any restrictions to use by non-academics:** none other than those specified by the licenses.

## Abbreviations

ANTLR: ANother Tool for Language Recognition
ChEBI: Chemical Entities of Biological Interest
EBI: European Bioinformatics Institute
GWT: Google Web Toolkit
IRI: International Resource Identifier
OWL: Web Ontology Language
RDF: Resource Description Framework
SDF: Structure-Data File
SPARQL: SPARQL Protocol and RDF Query Language
SQL: Structured Query Language
